# Copper-Triggered Aggregation of Ubiquitin

**DOI:** 10.1371/journal.pone.0007052

**Published:** 2009-09-16

**Authors:** Fabio Arnesano, Simone Scintilla, Vincenza Calò, Elena Bonfrate, Chiara Ingrosso, Maurizio Losacco, Teresa Pellegrino, Enrico Rizzarelli, Giovanni Natile

**Affiliations:** 1 Dipartimento Farmaco-Chimico, University of Bari “A. Moro”, Bari, Italy; 2 Consorzio Interuniversitario di Ricerca in Chimica dei Metalli nei Sistemi Biologici (CIRCMSB), Bari, Italy; 3 Dipartimento di Chimica, University of Bari “A. Moro”, Bari, Italy; 4 National Nanotechnology Laboratory of CNR-INFM and IIT Research Unit, University of Salento, Lecce, Italy; 5 Dipartimento di Scienze Chimiche, University of Catania, Catania, Italy; Mental Health Research Institute of Victoria, Australia

## Abstract

Neurodegenerative disorders share common features comprising aggregation of misfolded proteins, failure of the ubiquitin-proteasome system, and increased levels of metal ions in the brain. Protein aggregates within affected cells often contain ubiquitin, however no report has focused on the aggregation propensity of this protein. Recently it was shown that copper, differently from zinc, nickel, aluminum, or cadmium, compromises ubiquitin stability and binds to the N-terminus with 0.1 micromolar affinity. This paper addresses the role of copper upon ubiquitin aggregation. In water, incubation with Cu(II) leads to formation of spherical particles that can progress from dimers to larger conglomerates. These spherical oligomers are SDS-resistant and are destroyed upon Cu(II) chelation or reduction to Cu(I). In water/trifluoroethanol (80∶20, v/v), a mimic of the local decrease in dielectric constant experienced in proximity to a membrane surface, ubiquitin incubation with Cu(II) causes time-dependent changes in circular dichroism and Fourier-transform infrared spectra, indicative of increasing β-sheet content. Analysis by atomic force and transmission electron microscopy reveals, in the given order, formation of spherical particles consistent with the size of early oligomers detected by gel electrophoresis, clustering of these particles in straight and curved chains, formation of ring structures, growth of trigonal branches from the rings, coalescence of the trigonal branched structures in a network. Notably, none of these ubiquitin aggregates was positive to tests for amyloid and Cu(II) chelation or reduction produced aggregate disassembly. The early formed Cu(II)-stabilized spherical oligomers, when reconstituted in 1-palmitoyl-2-oleoyl-*sn*-glycero-3-phosphocholine (POPC) liposomes and in POPC planar bilayers, form annular and pore-like structures, respectively, which are common to several neurodegenerative disorders including Parkinson's, Alzheimer's, amyotrophic lateral sclerosis, and prion diseases, and have been proposed to be the primary toxic species. Susceptibility to aggregation of ubiquitin, as it emerges from the present study, may represent a potential risk factor for disease onset or progression while cells attempt to tag and process toxic substrates.

## Introduction

Failure to eliminate misfolded proteins can lead to the formation of toxic aggregates and cell death [1]. In eukaryotic cells, the Ubiquitin (Ub)–Proteasome System (UPS) is the main pathway for eliminating misfolded proteins [2]. Proteins are earmarked by the covalent attachment of a polyUb chain, which is specifically recognized by the proteasome. PolyUb chains can take on diverse structures depending on the lysine residue of Ub involved in chain elongation [3]. Although all seven surface lysine residues of Ub can be used in the formation of polyUb chains, Lys63- and Lys48-linked chains are the most frequently observed and the best characterized. Lys63-linked polyUb chains play a role in endocytosis, protein turnover, DNA damage tolerance, and in the inflammatory response through non-degradative signaling pathways. In contrast, Lys48-linked polyUb chains target their substrates to the proteasome for degradation [3].

Several neurodegenerative diseases are believed to share a common molecular mechanism involving protein misfolding and aggregation [4], [5] and an increasing number of observations indicate that metal ions are capable of accelerating these processes [6]–[9]. Biological investigations have demonstrated that exposure of the UPS to increasing amounts of metal ions affects its main proteolytic activity suggesting a close relationship between age-dependent rise of metal ions in the brain, UPS failure, and disease onset [10]. The link between UPS impairment and disease onset is also supported by the observation that protein aggregates within affected cells often contain Ub [11]–[14].

Lewy bodies are protein aggregates found in the cytoplasm of surviving dopaminergic neurons in Parkinson's disease [15]. The three main components of Lewy bodies are α-synuclein (α-syn), Ub, and parkin, a Ub ligase enzyme. These inclusions also contain other UPS components, molecular chaperones, and lipids. It is believed that accumulation of α-syn within intracellular inclusions is the primary event in Lewy body formation. Parkin and Ub are believed to be incorporated into the forming inclusion as they attempt to disaggregate it (secondary events). Exceptions to this sequence of events are seen in a rare form of juvenile PD found in Japan, where parkin aggregation is the initiating event, and a rare familial form of the disease involving Ub aggregation as the primary step. While α-syn aggregation has been extensively investigated and shown to be catalyzed by metal ions [16]–[18], no report has focused on the aggregation propensity of Ub, which has long been considered a very stable protein [19].

We have recently shown that Cu^II^ binds to Ub with 0.1 µM affinity and compromises protein stability [20]. The Cu^II^ affinity of Ub is comparable to that of amyloidogenic proteins involved in Parkinson's, Alzheimer's, and prion diseases [21], and of α-syn itself [22]. Differential Scanning Calorimetry (DSC) experiments carried out on Ub solutions with different metals (Cu^II^, Zn^II^, Ni^II^, Al^III^, and Cd^II^) indicate that only Cu^II^ has a specific negative effect on the thermal stability of Ub [20]. Since conditions that destabilize the native state of a protein render the macromolecular system more prone to aggregation, we decided to investigate whether the Cu^II^-induced alterations in Ub structure and stability may promote Ub aggregation and, thus, potentially compromise the protective function of the UPS in neurons. The present work demonstrates that Cu^II^ binding at specific sites and moderately low dielectric medium guide the protein through well-identified aggregation pathways.

## Results

### Cu^II^ binds at aggregation-prone regions of ubiquitin and causes protein destabilization

**Figure 1:**
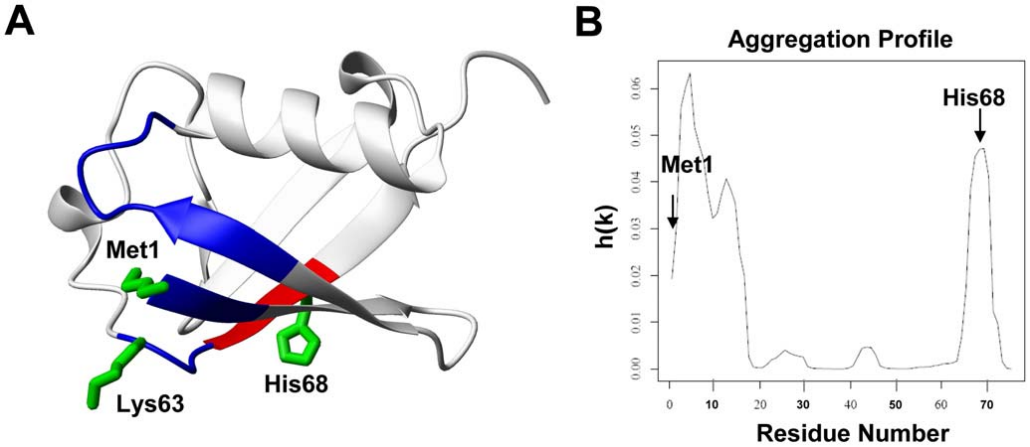
Cu^II^ ions target the aggregation-prone regions of Ub. A) Mapping the effects of paramagnetic Cu^II^ binding on the Ub NMR signals (PDB ID 1UBQ): Ub residues whose signals are broadened beyond detection are colored in blue for the primary site (Met1) and in red for the secondary site (His68); Lys63 is also shown; B) Aggregation profile of Ub obtained with the program PASTA.

In a previous investigation NMR experiments allowed us to identify two Cu^II^-binding sites on Ub: the first site is located at the N-terminus of the protein and involves the Met1 nitrogen and three oxygen donor ligands (residues 16–18) in a tetragonal arrangement, as confirmed by EPR spectra [20]; the second Cu^II^-anchoring site involves the imidazole nitrogen of His68 (Fig. 1A and supporting information Figure S1). Although far-UV circular dichroism (CD) spectra, recorded at 25°C, indicate very little overall change in Ub secondary structure upon Cu^II^ binding, thermal denaturation curves reveal a decrease of the unfolding temperature from 100°C for native Ub to 90°C for the Cu^II^-Ub system (supporting information Figure S2). By using the program PASTA (Prediction of Amyloid STructure Aggregation), which is based on a sequence-specific energy function derived from the propensity of two residues in globular proteins to be found paired in neighboring strands within a β-sheet [23] (Fig. 1B), we noticed that the two Cu^II^ binding regions are predicted to be the most aggregation-prone for Ub, thus we suspected that interaction with Cu^II^ could be a key factor promoting Ub aggregation.

### Cu^II^-induced self-oligomerization of ubiquitin

**Figure 2:**
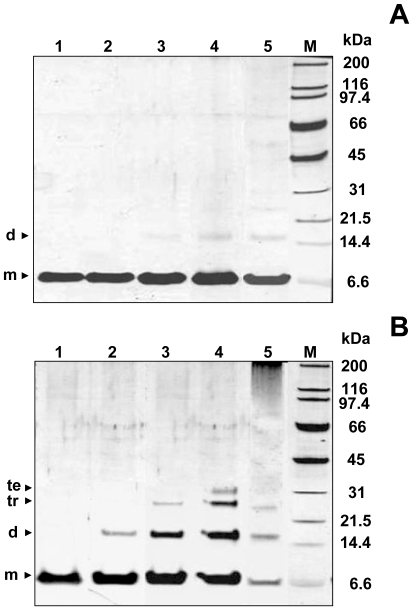
Analysis of Cu^II^-stabilized Ub oligomers. SDS-PAGE analysis of Ub incubated at 37°C in the absence of Cu^II^ (A) and with 3 mol equiv of Cu^II^ (B) at different incubation time intervals: 2 h (*lane 1*), 3 d (*lane 2*), 1 w (*lane 3*), 2 w (*lane 4*), 2 mo (*lane 5*).

**Figure 3:**
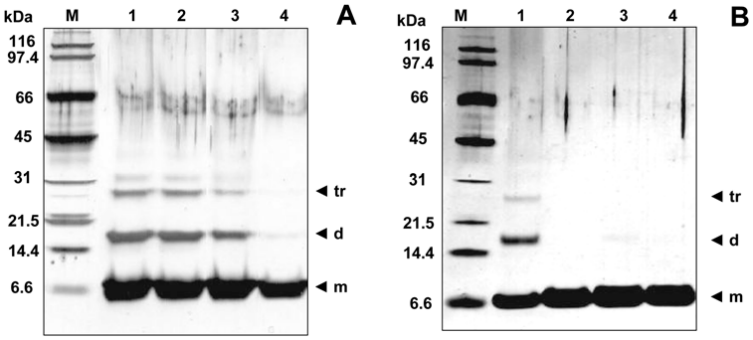
Disruption of Cu^II^-stabilized Ub oligomers by addition of chelating agents. (A) SDS-PAGE of Ub incubated for 2 weeks at 37°C with 3 mol equiv of Cu^II^ in 20% TFE (*lane 1*) and then incubated for 2 h with increasing concentrations of EDTA: 100 µM (*lane 2*), 200 µM (*lane 2*), and 300 µM (*lane 4*); (B) SDS-PAGE of Ub incubated for 2 weeks at 37°C with 3 mol equiv of Cu^II^ in 20% TFE (*lane 1*) and then incubated for 2 h with 300 µM IDA (*lane 3*). Control experiments performed on Ub incubated in water (*lane 2*) or in 20% TFE (*lane 4*) are also reported.

The aggregation of Ub induced by Cu^II^ was monitored by sodium dodecyl sulfate-polyacrylamide gel electrophoresis (SDS-PAGE). SDS-PAGE of control reactions lacking Cu^II^ ions and incubated at 37°C shows a single band at ∼8 kDa, corresponding to the apparent molecular weight of monomeric Ub (Fig. 2A). By contrast, SDS-PAGE of Ub incubated with three mol equivalents of Cu^II^ at 37°C, shows a time-dependent appearance of discrete bands at ∼16 kDa, ∼24 kDa, and ∼32 kDa corresponding to SDS-resistant Ub dimer, trimer, and tetramer, respectively. After two months of incubation, Ub oligomers are reduced and larger ubiquitin aggregates, unable to enter the separating gel, are formed (Fig. 2B). The electrophoretic behavior is similar when the Cu^II^ concentration is varied in the range 10–100 µM (corresponding to 0.3−3 mol equivalents of Cu^II^ to Ub), the rate and the extent of aggregation increasing as a function of Cu^II^ concentration. The bands corresponding to Ub oligomers disappear upon Cu^II^ chelation by ethylenediaminetetraacetic acid (EDTA) or iminodiacetic acid (IDA), a tetra- and a tridentate ligand, respectively (Fig. 3A,B), clearly indicating that Cu^II^ ion is responsible for oligomer formation. Preliminary experiments using iron and calcium, two metal ions correlated with Parkinson's disease and other neurodegenerative disorders, indicate no formation of SDS-resistant oligomers after two weeks time (supporting information Figure S3).

**Figure 4:**
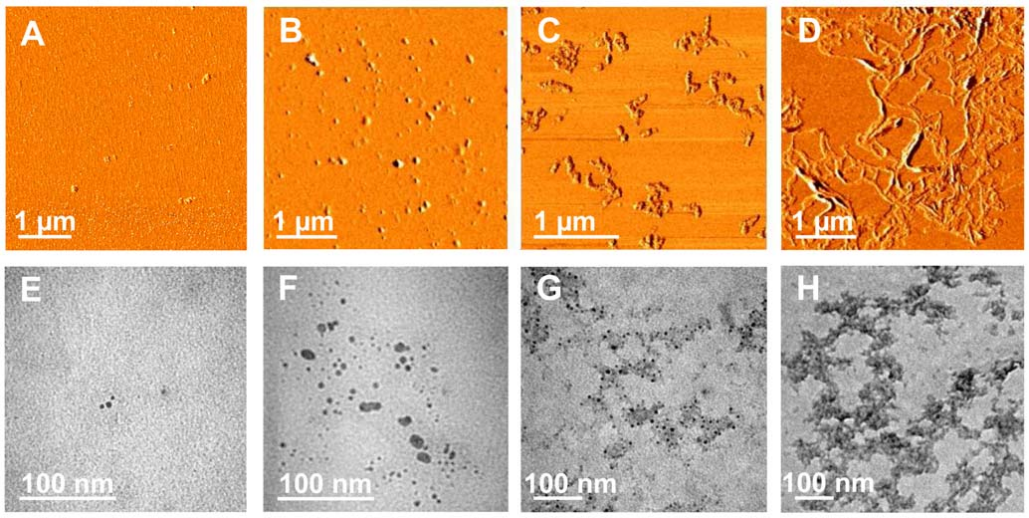
Ub aggregate morphologies for long-term incubations with Cu^II^ and/or TFE. Phase-mode AFM images (A, B, C, D) and TEM micrographs (E, F, G, H) of Ub structures after two months of incubation at 37°C in the absence of Cu^II^ (A and E); with 3 mol equiv of Cu^II^ (B and F); with 20% TFE (C and G); with 3 mol equiv of Cu^II^ in 20% TFE (D and H).

No significant change in the CD profile was observed for Ub incubated with three mol equivalents of Cu^II^ over a period of two months (supporting information Figure S4). Consistently, attenuated total reflectance-Fourier transform infrared (ATR-FTIR) spectra did not reveal any significant variation in the amide I band, which in native Ub falls at 1642 cm^−1^. For the same two months of incubation of Ub with Cu^II^ at 37°C, atomic force microscopy (AFM) and transmission electron microscopy (TEM) reveal the presence of clusters of spherical oligomers of different sizes ranging from 5 to 25 nm (Fig. 4B,F). It is worth recalling that these spherical oligomers are SDS-resistant and are destroyed upon Cu^II^ chelation. By comparison, after two months of incubation of Ub in water in the absence of Cu^II^, protein aggregates are nearly absent in AFM and TEM images (Fig. 4A,E).

### Influence of solvent polarity upon ubiquitin aggregation

The addition of a moderate amount (20%, v/v) of 2,2,2-trifluoroethanol (TFE) is commonly used to mimic the local decrease of dielectric constant experienced in the proximity of a membrane surface, estimated to be half the dielectric constant in “bulk” water [24]. Addition of TFE has been shown to influence the structure and kinetics of fibrillation of α-syn [24] as well as of other amyloidogenic proteins [25], [26], possibly by decreasing the solvent polarity and strengthening the protein-protein hydrogen bond interactions. In the absence of Cu^II^, incubation of Ub with 20% TFE over a period of two months did not show any significant change in the CD profile or any variation in the amide I region as revealed by ATR-FTIR. AFM revealed the formation of short beaded chains, each composed of spherical subunits of 6–8 nm height (Fig. 4C,G). SDS-PAGE showed that, unlike the spherical oligomers formed in the presence of Cu^II^, these aggregates are not SDS-resistant and migrate as monomers on the gel.

### Remarkable cooperativity between Cu^II^ and solvent polarity

**Figure 5:**
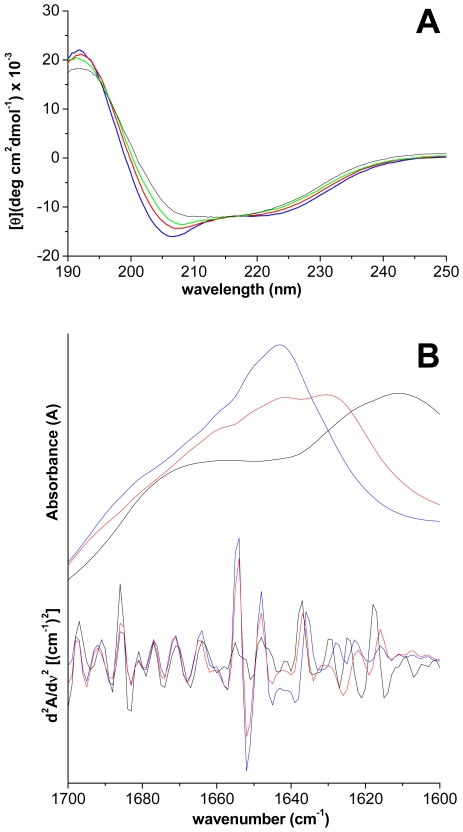
Cu^II^ and TFE induced conformational change of Ub. A) Far-UV CD spectra of Ub incubated at 37°C with 3 mol equiv of Cu^II^ in 20% TFE for different time intervals: 1 h (blue line); 5 d (red); 1 mo (green); 2 mo (black); B) ATR-FTIR spectrum (absorbance at the top and second derivative at the bottom) in the amide I region of native Ub (blue line) and of Ub incubated with 3 mol equiv of Cu^II^ in 20% TFE for 1 w (red) and 2 mo (black).

A change in the CD profile over time was instead observed for Ub incubated with Cu^II^ in water with 20% TFE (Fig. 5A). In particular, the negative bands with minima at ∼206 and ∼222 nm gradually disappear merging in a single broad band centered at 210–215 nm, in accord with a higher β-sheet content. This change in secondary structure was confirmed by inspection of the amide I region in ATR-FTIR spectra (Fig. 5B). The appearance of a new band at 1630 cm^−1^, whose intensity increases with time, reveals an increase of the β-sheet content, most likely stemming from intermolecular interactions. After two months the amide I band shifts at 1610 cm^−1^, indicative of β-sheet structures with stronger intermolecular hydrogen bonds.

**Figure 6:**
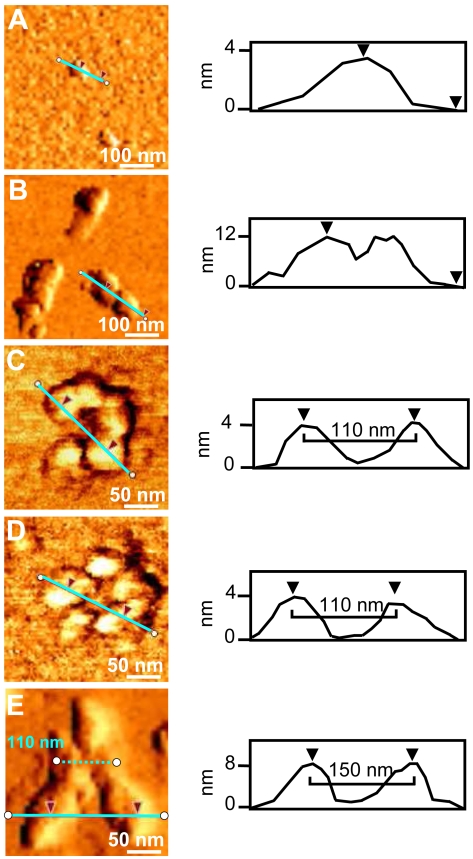
Hierarchical assembly of Ub aggregates. Phase-mode AFM images of Ub structures after incubation at 37°C with 3 mol equiv of Cu^II^ in 20% TFE for different time intervals: 1 d (A); 5 d (B); 10 d (C, D); 1 mo (E). (*Right panel*) Cross-sectional profile, taken along the cyan line, of the corresponding topographic image.

AFM shows the rapid formation of spherical particles with heights comprised between 2 and 8 nm (mean height 4±1.6 nm, Fig. 6A), consistent with the size of the Ub oligomers detected by SDS-PAGE. After 4–5 days of incubation at 37°C, straight and curved chains of 8–12 nm height and 200–300 nm length are observed (Fig. 6B). Discrete annular oligomeric species appear after 10 days of incubation. When imaged at higher magnification, these annular species appear to be composed of 5 or 6 spherical particles of 3–5 nm height, that form open and closed ring-like structures with a diameter of ∼110 nm, as estimated by measuring the peak-to-peak distance of a cross-section (Fig. 6C,D). The similar heights of the spheres and of the ring structures suggest that the spherical particles are most likely the precursors of the annular species. In four weeks time, straight filaments of 6–8 nm height and 90–150 nm length grow radially from the periphery of the central ring forming trigonal branched structures (Fig. 6E). The cross-sectional analysis reveals that the central ring almost preserves the diameter of ∼110 nm (dotted line in Fig. 6E). After two months the trigonal branched structures interconnect in an extended network of filaments (Fig. 4D,H). Although complex, the network appears to be formed of a variety of geometries generated by the random coalescence of trigonal branched structures, whose central rings act as network nodes. Amorphous aggregates were detected only occasionally and in negligible quantity.

Importantly, addition of Cu^II^ to Ub incubated for two months with 20% TFE alone or addition of TFE to Ub incubated for two months with three mol equivalents of Cu^II^ alone, induces a rapid process of convergence leading in about one week to the same endpoint of the three-component mixture, i.e. CD spectra indicative of a β structure and AFM showing an extended filament network.

Two dyes are commonly used to reveal amyloid fibrils: Thioflavin T (ThT), a benzothiazole that exhibits enhanced fluorescence upon binding to amyloid fibrils, and 1-anilinonaphthalene-8-sulfonate (ANS), a naphthalene derivative whose interaction with hydrophobic sites causes an increase in fluorescence and a blue shift of the peak maximum [27]. Notably, neither ThT nor ANS are sensitive to any of the Ub aggregates obtained under the above conditions (data not reported) indicating that no amyloid is formed. Negative response to tests for amyloid has also been obtained for aggregates of the yeast prion determinant Sup35 which leads to non-fibrillar *filaments* [28].

**Figure 7:**
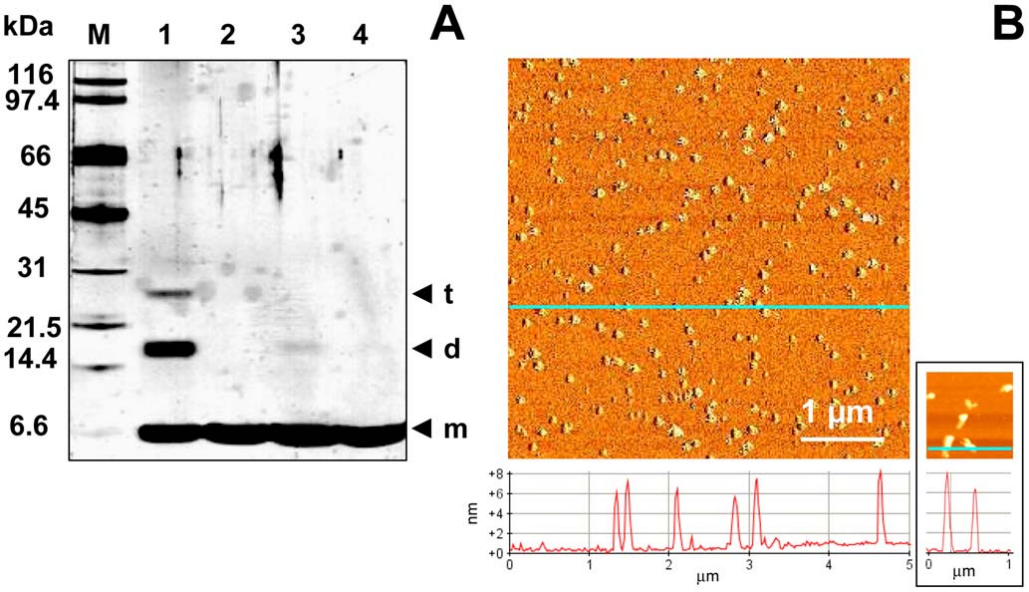
Redox-mediated aggregate disassembly. A) SDS-PAGE of Ub incubated for 2 weeks at 37°C with 3 mol equiv of Cu^II^ in 20% TFE before (*lane 1*) and after (*lane 3*) 2 h treatment with ascorbic acid. Control experiments performed on Ub incubated in water (*lane 2*) or in 20% TFE (*lane 4*) are also reported; B) Phase-mode AFM image and cross-sectional profile, taken along the cyan line, of Ub incubated for 2 months at 37°C with 3 mol equiv of Cu^II^ in 20% TFE after 2 h treatment with ascorbic acid. The *inset* at the bottom right shows an AFM image and a cross-sectional profile of Ub incubated for 2 weeks at 37°C in 20% TFE.

AFM also shows that the extended aggregation networks formed in aged samples of Ub incubated with Cu^II^ in 20% TFE are disrupted by Cu^II^ reduction, yielding a homogeneous population of spherical particles of 6–8 nm height which are not SDS-resistant (Fig. 7). The height of the spherical particles and the behavior in SDS-PAGE (non-resistant), make them more similar to the individual particles formed in TFE alone (Fig. 7, *inset*).

### Cu^II^ and TFE cooperativity detected at the molecular level

**Figure 8:**
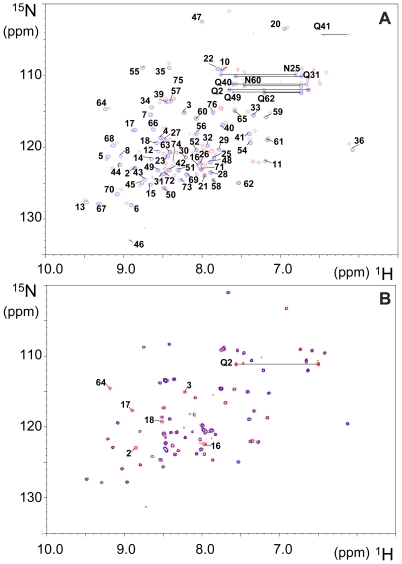
Paramagnetic Cu^II^ broadening effects in NMR spectra of Ub in TFE. A) Overlay of ^1^H,^15^N HSQC spectra of Ub in 50 mM ammonium acetate buffer at pH 6.5 (blue contours) and Ub in the same buffer with 20% TFE (red contours). Backbone amide cross-peaks and side-chains of Gln/Asn are labeled; B) Overlay of ^1^H,^15^N HSQC spectra of Ub in 50 mM ammonium acetate buffer at pH 6.5 with 20% TFE before (red contours) and after (blue contours) addition of 0.1 mol equiv of Cu^II^. Cross-peaks that disappear after Cu^II^ addition are labeled.

The ^1^H,^15^N heteronuclear single quantum coherence (HSQC) spectrum of Ub was recorded in the presence of 20% TFE (Fig. 8A). With respect to pure water, the addition of the organic solvent produces significant chemical shift changes in most of the amide resonances, the most affected ones corresponding to the β-strand regions of the protein. These changes are likely to be a consequence of the strengthening of existing hydrogen bonds and salt bridges while there is no sign, whatsoever, of denaturation.

Addition of substoichiometric amounts of Cu^II^ to Ub in 20% TFE (0.1∶1 Cu^II^ to Ub ratio) results in the disappearance of a number of amide cross-peaks in the ^1^H,^15^N HSQC spectrum (Fig. 8B). Affected residues are confined to a well-defined region at the N-terminus of the protein, which represents the primary Cu^II^ binding site (Fig. 1A). Notably, the backbone resonances of residues 1–3, while experiencing very little or no shift in 20% TFE (Fig. 8A) even after a few weeks of incubation (indicating that TFE alone does not alter the conformation of the N-terminus of Ub), undergo the largest broadening upon addition of Cu^II^. Further addition of Cu^II^ (0.5∶1 Cu^II^ to Ub ratio) causes extensive broadening of all Ub resonances, as expected for the formation of aggregates. This was not the case in pure water where up to 3∶1 Cu^II^ to Ub ratio there was broadening of Ub residues only close to the major (N-terminus) and minor (His68) binding sites, clearly indicating that in pure water the onset of the Cu^II^-induced aggregation is delayed [20].

### Cu^II^-stabilized oligomers of ubiquitin form pores in lipid bilayers

**Figure 9:**
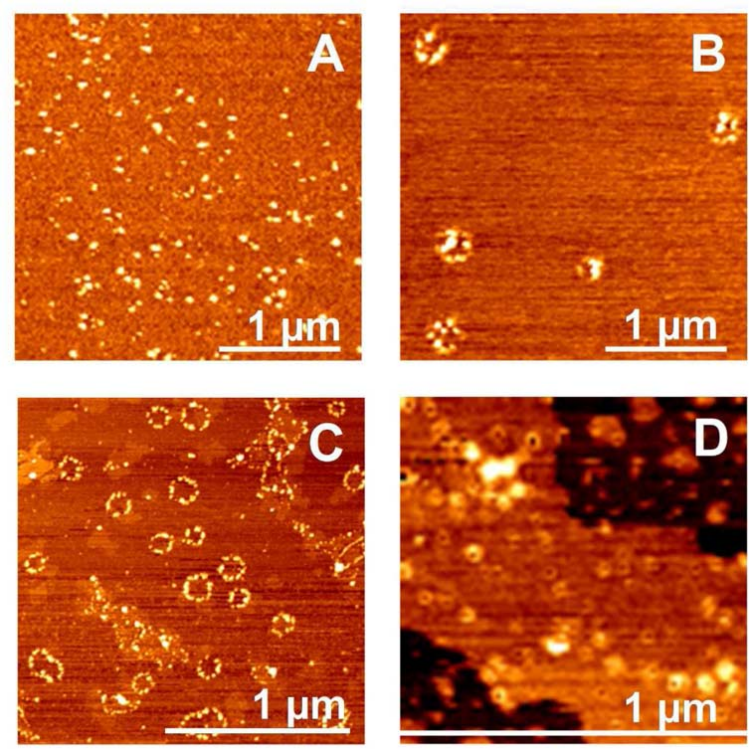
Annular and pore-like assemblies of Cu^II^-stabilized Ub oligomers. Topographic AFM images of Ub preincubated for two weeks at 37°C with 3 mol equiv of Cu^II^ in aqueous solution (A) and then dissolved in 20% TFE (B), in POPC liposomes (C), or in POPC planar bilayers (D).

pone.0007052.When .tifpreformed Cu^II^-stabilized spherical oligomers of Ub (mean height of 4 nm, Fig. 9A) are dissolved in water containing 20% TFE, formation of supramolecular annular assemblies with diameters comprised between 100 and 200 nm is observed (Fig. 9B). Although water-TFE mixtures can reasonably model the decrease of dielectric constant in the proximity of a membrane surface, a much better mimic of the amphiphilic membrane environment can be provided by phospholipid bilayers. Thus, we prepared liposomes of 1-palmitoyl-2-oleoyl-*sn*-glycero-3-phosphocholine (POPC), as described in *Materials and Methods*, and treated them with preformed Cu^II^-stabilized oligomers of Ub. AFM revealed the formation of annular assemblies entirely similar to those observed in water containing 20% TFE (Fig. 9C). In both cases the supramolecular rearrangement can be ascribed to a decrease in dielectric constant of the medium. If liposomes are deposited on freshly cleaved mica and allowed to fuse and rupture upon contact with the mica surface for 20 min, planar lipid bilayers with thickness of 5.0–5.5 nm are formed (Figure S5 A,B). Applying this procedure (20 min delay after deposition on freshly cleaved mica surface) to liposomes treated with preformed Cu^II^-stabilized oligomers of Ub, membrane-inserted pore-like structures with an outer diameter of only 20–25 nm were formed (Fig. 9D). These structures protrude above the surrounding flat lipid bilayer (Figure S5 C,D). Annular and pore-like structures are not observed when Cu^II^-stabilized oligomers of Ub are pretreated with EDTA, which has been shown to destroy the Ub oligomers formed by incubation with Cu^II^.

## Discussion

The cellular concentrations of the two forms of ubiquitin, free Ub and polyUb chains, are closely interconnected and may change upon various cellular events; for instance heat stress induces an increase in polyUb chains at the expenses of free Ub [29]. Decreased Ub availability in neurons is sufficient to cause neuronal dysfunction and death [30]. On the other hand, increased Ub concentrations in cerebrospinal fluids of patients affected by neurodegenerative diseases and amyloidosis may have a neuroprotective effect [31].

The presence of Ub within inclusion bodies was noted in a large variety of neurodegenerative diseases [11]–[14], suggesting that disruption of Ub homeostasis may be a common factor in the pathogenesis of these disorders; however, Ub has never been considered to be susceptible to aggregation. Here, we show that Cu^II^ ions target the two predicted aggregation-prone regions of Ub and induce protein oligomerization. Three experiments were performed. i) In pure water, incubation with Cu^II^ leads to formation of Ub oligomers, which appear as spherical particles that can progress from dimers to large Ub conglomerates and which are SDS-resistant. ii) In the absence of copper, but in a solvent of lower dielectric constant (water containing 20% TFE), beaded chains composed of spherical subunits are formed after weeks of incubation; these spherical particles, however, are not SDS-resistant. iii) Finally, the simultaneous presence of Cu^II^ ions and of a medium of lower dielectric constant leads initially to the formation of spherical particles (SDS-resistant oligomers), followed by clustering of these particles in annular species, formation of trigonal branched structures growing radially from the annular species, and finally interconnection of these branched structures in filament networks. Notably, the Ub aggregates obtained under the above conditions are negative to ThT and ANS tests for amyloid. Although negative to the ThT test, the Ub aggregates formed in iii) appear to contain an intermolecular β-sheet structure, as determined by far-UV CD and ATR-FTIR spectroscopy (Fig. 5). Significantly, the amide I band initially shifts to a value borderline between the typical range of globular β-sheet proteins (1645–1630 cm^−1^) and that of amyloid fibrils (1630–1610 cm^−1^) [32]. The negativity to the ANS test indicates that the oligomerization/aggregation process takes place without exposing extensive hydrophobic patches. These results suggest that in our case the spherical and annular oligomers do not function as precursors of amyloid fibrils, but lead to particular filament networks which are negative to ThT and ANS tests. The spherical oligomeric particles formed in i) and iii) appear to be stabilized by intermolecular covalent-type interactions that make them SDS-resistant. Mass spectral data do not show sign of protein oxidation over the course of incubations, suggesting that SDS-resistant oligomers are stabilized by Cu^II^ coordination rather than by oxidative cross-link formation. Indeed, Cu^II^ removal by chelation (Fig. 3) or reduction to Cu^I^ (Fig. 7) produces oligomer disappearance from the gels and disassembly of the filament networks into spherical particles of the type observed in ii), which are sensitive to SDS-PAGE and migrate as monomers.

The aggregation process of Ub closely matches, in terms of size and morphology of intermediate species, those described for α-syn incubated with Ca^II^ ions [33] or undergoing oxidation induced by Fe^III^ ions in dithiothreitol [34]. In both studies, α-syn was shown to form spherical and annular oligomers that might represent potentially toxic *protofibrils* [35], [36]. The intermediate annular species, common to several neurodegenerative disorders, appear to be the most cytotoxic *in vivo* by forming pore-like structures which cause membrane permeabilization and disruption of ion homeostasis [37]. Indeed, kinetic stabilization of α-syn protofibrils, under conditions inhibiting their conversion to mature fibrils, enhances the harmful effects of aggregation and accelerates disease progression [38]–[41]. Moreover, UPS impairment correlates with the appearance of smaller intermediate forms of protein aggregates rather than with the formation of inclusion bodies [42]. Cu^II^-stabilized spherical oligomers of Ub form annular structures in intact liposomes as a consequence of decrease in dielectric constant in proximity to a membrane surface. When liposomes are allowed to fuse and rupture on the mica surface to form a planar phospholipid bilayer, the Ub oligomers form compact, pore-like structures penetrating into the membrane (Fig. 9).

Moving from micro- to nanoscale, NMR data suggest that Cu^II^ coordination to Ub may destabilize the protein starting from the N-terminal region, which represents the primary Cu^II^ binding site. Cu^II^ coordination to the N-terminus is emerging as a common feature of several amyloid-related proteins [21], including α-syn [43]. In the native Ub structure, the first residue, Met1, is involved in two key hydrogen bonds [44]: one between the amino-terminus and the CO of Val17 and the other between the side chain sulfur and the amide NH of Lys63 (Fig. 1A). Thus, Cu^II^ binding to Ub could compromise the autophagic clearance of protein inclusions, a process regulated by Lys63-linked polyubiquitination [45].

Cu^II^ appears to play a unique role in Ub destabilization and aggregation. Other metal ions (Zn^II^, Ni^II^, Al^III^, and Cd^II^) do not destabilize Ub and Cu^II^ reduction to Cu^I^ offsets Ub destabilization and aggregation. Copper is generally believed to be transported in the cells by the plasma membrane permease Ctr1 in the +1 oxidation state; it is yet unproven, however, that Cu^I^ is the only permeant species [46]. There is unequivocal evidence that Ctr1 is not the only protein capable of mediating copper entry into mammalian cells, and it is quite possible that Cu^II^ rather than Cu^I^ is transported by Ctr1-independent mechanisms, based on divalent metal ion inhibition [47], [48]. Consistent with the reducing environment of the cytosol, X-ray absorption spectroscopy indicates the presence of low-coordinate monovalent Cu^I^ in this compartment [49]–[51]; however, Cu^II^ appears to be abundant inside both normal and Parkinson's disease neurons of *substantia nigra* [52]. That Cu^II^ could gain access to special intracellular districts of normal or diseased cells is also supported by the recent identification of the Fre6 vacuolar metalloreductase [53], and in neurodegenerative diseases the intravesicular material undergoes ubiquitination [11], [54].

Importantly, it has recently been reported that extracellular aggregates, including the Alzheimer'disease amyloids, can be internalized by mammalian cells, gain access to the cytosol, and colocalize with Ub and UPS components [55]. These new findings strongly argue that Ub may come in contact, in the cytosol, with extracellular aggregates enriched with oxidized Cu^II^. Elemental mapping of amyloid deposits of Alzheimer'disease brain revealed ‘hot spots’ of accumulated metal ions, particularly copper and zinc [56], [57]. Moreover, an increment of copper density was observed in brain tissues that were positively stained for Ub [58], and Alzheimer'disease amyloid plaques can be dissolved by Cu^II^ chelators [59].

Therefore, the interaction of Ub with Cu^II^ could be a pathological event taking place inside intracellular organelles or in the cytosolic compartment of cells which attempt to tag and process toxic substrates. Oxidative stress, membrane breaching, abnormal metal ion homeostasis, and metal miscompartmentalization can foster this process [60], [61]. In addition, factors which favor protein and metal desolvation (e.g. lower dielectric constant near a membrane surface or in the proximity of inclusion bodies) [62] may significantly increase the Cu^II^ binding affinity and aggregation propensity of Ub. Lipids and vesicle membranes were found in Lewy bodies on autopsy [63] and lipid-bound soluble cytosolic oligomers were increased in brain extracts from patients with Parkinson's disease or dementia with Lewy bodies [64].

Ub has been widely used as model for stability, folding, and structural studies [19]. The present results disclose new possible connections and research perspectives for this intriguing protein.

## Materials and Methods

### Sample preparation and aggregation assays

Human unlabeled and isotopically labeled Ub (Ubiquilabel™ ^15^N, 98%) were purchased from VLI Research Inc. 2,2,2-trifluoroethanol (TFE) was purchased from Sigma-Aldrich. The lyophilized protein was dissolved in 5 mM ammonium acetate buffer (pH 6.5). Aggregation was initiated by incubating aliquots of the protein at a concentration of 0.3 mg mL^−1^ (35 µM) at 37°C with 0.3 to 3 mol equivalents of Cu^II^, added as acetate salt Cu(OAc)_2_. Long-term incubations were conducted at 37°C in the absence and in the presence of three mol equivalents of Cu^II^, and with or without 20% (v/v) TFE. All the incubations were performed under sterile conditions (i.e., all glassware and cuvettes were sterilized and all buffers were filtered through 0.02 µm Whatman Anotop filters). All solutions were prepared by using ultrapure Milli-Q water from Millipore purification system. The volume used for each incubation experiment was at least 100 µL and each experiment was performed at least in triplicate.

### Preparation of liposomes and phospholipid bilayers

1-palmitoyl-2-oleoyl-*sn*-glycero-3-phosphocholine (POPC) was dissolved in chloroform and dried under a flow of dry N_2_. The thin film of POPC was dissolved in 0.5 mL of Na-cholate (4% solution) in phosphate buffer (50 mM, KCl 100 mM, pH 6.8). The solution was sonicated in ice bath for a few seconds with a titanium tip and loaded on a Sephadex G-50 superfine gel filtration column previously equilibrated with phosphate buffer. During the elution the mixed micelles are deprived of the detergent; meanwhile, phospholipids rearrange to form liposomes whose diameter was estimated to be ∼20 nm using dynamic light scattering. Oligomers of Ub incubated at 35 µM concentration with three mol equivalents of Cu^II^ for 96 h were mixed with the POPC liposomes at a 1∶20 protein∶lipid weight ratio. Liposomes reconstituted with Ub oligomers were then deposited on freshly cleaved mica for 20 min and allowed to fuse and rupture upon contact with the mica surface forming planar lipid bilayers, as previously described [65].

### Far-UV Circular Dichroism

CD spectra were recorded on a Jasco J-810 spectropolarimeter at 35 µM Ub concentration by using a quartz cuvette with a 0.1 cm optical path, a wavelength interval of 185–250 nm and 0.1 nm data pitch. All spectra, corresponding to an average of 5 scans, were baseline corrected and then smoothed by applying adjacent averaging or an FFT filter. The ellipticity is reported as mean residue molar ellipticity (deg cm^2^ dmol^−1^) according to [θ] = 100·[θ]_obs_/(*C*·*L*·*N*), where [θ]_obs_ is the observed ellipticity in degrees, *C* is the molar concentration of the protein, *L* is the optical path length (in cm), and *N* is the number of aminoacids (*N* = 76 for Ub).

### Attenuated Total Reflectance-Infrared Spectroscopy

FTIR spectra were recorded using a Perkin-Elmer Spectrum One spectrometer equipped with a liquid N_2_-cooled MCT detector in ATR mode, and purged with a continuous flow of N_2_ gas. Typically, 64 interferograms were acquired at 4 cm^−1^ resolution. Spectra were measured at 25°C for samples containing 6.0 mg mL^−1^ Ub in 15 mM 2-(*N*-morpholino)ethanesulfonic acid (MES) buffer (pH 6.5) in the absence and in the presence of three mol equivalents of Cu^II^, and with or without 20% (v/v) TFE. An aliquot of the solution (3 µL) was loaded on the surface of a three-reflection diamond prism (3 mm diameter) of the ATR accessory (Sens*IR* technologies, UK). The sample was dried under N_2_ gas flow. For each sample, the corresponding buffer spectra were subtracted. Spectra were normalized according to the maximum intensity of amide A band (∼3300 cm^−1^). Data analysis was performed using second-derivative to identify component band positions.

### Nuclear Magnetic Resonance Spectroscopy

NMR experiments were performed on a 350 µM ^15^N-enriched sample of Ub in 50 mM ammonium acetate buffer (pH 6.5) at 25°C in 90% H_2_O and 10% D_2_O and in 20% TFE, 70% H_2_O and 10% D_2_O. Resonance assignment of the apoprotein was carried out by using available ^1^H and ^15^N chemical shift data [66], [67], with the aid of 2D TOCSY and NOESY, and 3D ^15^N-edited NOESY spectra. The titration of Ub with Cu^II^ with or without 20% (v/v) TFE was followed by ^1^H,^15^N HSQC [68], [69]. All spectra were collected on a Bruker Avance 600 with an UltraShield Plus magnet using a triple-resonance probe equipped with z axis self-shielded gradient coils, processed using the standard Bruker software (TOPSPIN), and analyzed through the programs CARA (The Computer Aided Resonance Assignment Tutorial, R. Keller, 2004, CANTINA Verlag), developed at ETH-Zürich, and SPARKY 3 (T. D. Goddard and D. G. Kneller, University of California, San Francisco). Cross-peaks affected during Cu^II^ titration were identified by comparing their intensities (*I*) with those of the same cross-peaks (*I_0_*) in the data set of samples lacking Cu^II^. The *I*/*I_0_* ratios were plotted as a function of the protein sequence to obtain intensity profiles.

### Gel electrophoresis

The time-dependent self-oligomerization of Ub was evaluated by Tris-Tricine/SDS-PAGE silver stained gels, as reported for α-syn [8]. Briefly, Ub (35 µM) was incubated at 37°C with Cu(OAc)_2_ (0.3, 1, and 3 mol equivalents to Ub) in 20 mM MES, pH 6.5, with or without 20% (v/v) TFE. Aliquots of the reaction mixture were drawn at different time intervals and kept for 1 h at 37°C in the presence of 0.3 mM *N*-(ethoxycarbonyl)-2-ethoxy-1,2-dihydroquinoline (EEDQ) coupling reagent, prepared as a 3 mM stock solution in DMSO. Chemical cross-linking was stopped by mixing the sample with an equal volume of buffer consisting of 8% (w/v) SDS, 24% (v/v) glycerol, 0.015% Coomassie Blue G in 0.9 M Tris-HCl, pH 8.45, followed by boiling for 5 min. Samples were loaded on triphasic discontinuous polyacrylamide gels (5%, 10%, 16.5% w/v) and run for 180 min at 25 mA. The ladder formation was revealed with the silver staining procedure [70]. To test the SDS-resistance of Ub oligomers the experiments were repeated avoiding the step of coupling with EEDQ. To test the role of Cu^II^ on oligomer stability, protein mixtures were further incubated for 2 h in the presence of up to ten-fold excess of ethylenediaminetetraacetic acid (EDTA), iminodiacetic acid (IDA), or ascorbic acid.

### Dynamic Light Scattering

Size distribution by intensity of the scattered light was obtained using a Zetasizer Nano ZS dynamic light scattering device from Malvern Instruments. The temperature was maintained at 37°C by a thermostating system. The DTS0112 low-volume disposable cuvettes (pathlength 1 cm) were used. The solutions were filtered immediately before use to eliminate any impurity using Whatman Anotop 10 syringe filters with 0.02 µm pore size.

### Transmission Electron Microscopy

Electron micrographs were acquired with a Jeol JEM 1011 microscope operated at an acceleration voltage of 100 kV. Aliquots of 10 µL of the sample were placed on a carbon-coated copper grid (Formvar/Carbon 300 Mesh Cu) and negatively stained with 2% aqueous phosphotungstic acid (pH adjusted to 6.5 using NaOH).

### Atomic Force Microscopy

AFM investigations were performed using a PSIA XE-100 SPM system operating in tapping mode in air by using a silicon nitride tip (model MLCT-AUHW, Park Scientific) with a cantilever having a spring constant of 0.01 N m^−1^. Incubations were stirred gently and aliquots of 3 µL were cast on the surface of freshly cleaved mica (NanoAndMore GmbH). The deposited samples were left to dry in air at room temperature for 2 min, rinsed with MilliQ water and gently dried with N_2_ flow. Micrographs were collected simultaneously both in height and phase mode by sampling the surface at a scan rate ranging between 1 and 0.5 Hz at a resolution of 512×512 pixels. The images were analyzed by using XEI software and topographic images were plane-adjusted.

## Supporting Information

Figure S1 Paramagnetic Cu(II) broadening effects in NMR spectra of Ub. Overlay of 1H,15N HSQC spectra of Ub in 50 mM ammonium acetate buffer at pH 6.5 with one (red contours) and three (blue contours) mol equiv of Cu(II). Backbone amide cross-peaks and side-chains of Gln/Asn residues that disappear upon addition of one equiv of Cu(II) are indicated with circles and labeled in blue; cross-peaks that disappear after addition of three equiv of Cu(II) are labeled in red.
(0.29 MB TIF)

Figure S2 Effect of Cu(II) on thermal denaturation of Ub. Far-UV CD spectra of Ub in the absence (A) and in the presence (B) of one mol equiv of Cu(II) recorded at increasing temperatures from 25 to 105°C in steps of 5°C. The plot of molar ellipticity at 220 nm vs. temperature is shown in (C) for Ub in the absence (red curve) and in the presence of Cu(II) (black curve).
(0.43 MB TIF)

Figure S3 Comparing the effect of Fe(II), Ca(II), and Cu(II) on Ub oligomerization. SDS-PAGE of Ub incubated for 2 weeks at 37°C with 3 mol equiv of Fe(II) (lane 2), Ca(II) (lane 3), and Cu(II) (lane 4). Control experiments were performed on Ub incubated for 2 weeks at 37°C in water in the absence of metal ions (lane 1) and on hen egg white lysozyme incubated for 2 weeks at 37°C with 3 mol equiv of Cu(II) (lane 5).
(0.43 MB TIF)

Figure S4 Effect of Cu(II) on secondary structure of Ub. Far-UV CD spectra of Ub incubated at 37°C with 3 mol equiv of Cu(II). Spectra were recorded at different incubation times over a period of two months.
(0.28 MB TIF)

Figure S5 Reconstitution of Cu(II)-stabilized Ub oligomers in phospholipid bilayers. Phase-mode AFM images and cross-sectional profile, taken along the red line, of POPC liposomes (A) and POPC planar bilayers (B) in the absence of protein; topographic AFM images of annular (C) and pore-like structures (D) formed by Ub preincubated for two weeks at 37°C with 3 mol equiv of Cu(II) in aqueous solution and then dissolved in POPC liposomes and in POPC planar bilayers. The corresponding cross-sectional profiles and 3D views are shown at the bottom.
(3.31 MB TIF)
