# Supplementary figures and images for: Copper-Triggered Aggregation of Ubiquitin
Source: PLoS One. 2009 Sep 16;4(9):e7052. doi: 10.1371/journal.pone.0007052 (PMC2737635; doi:10.1371/journal.pone.0007052)

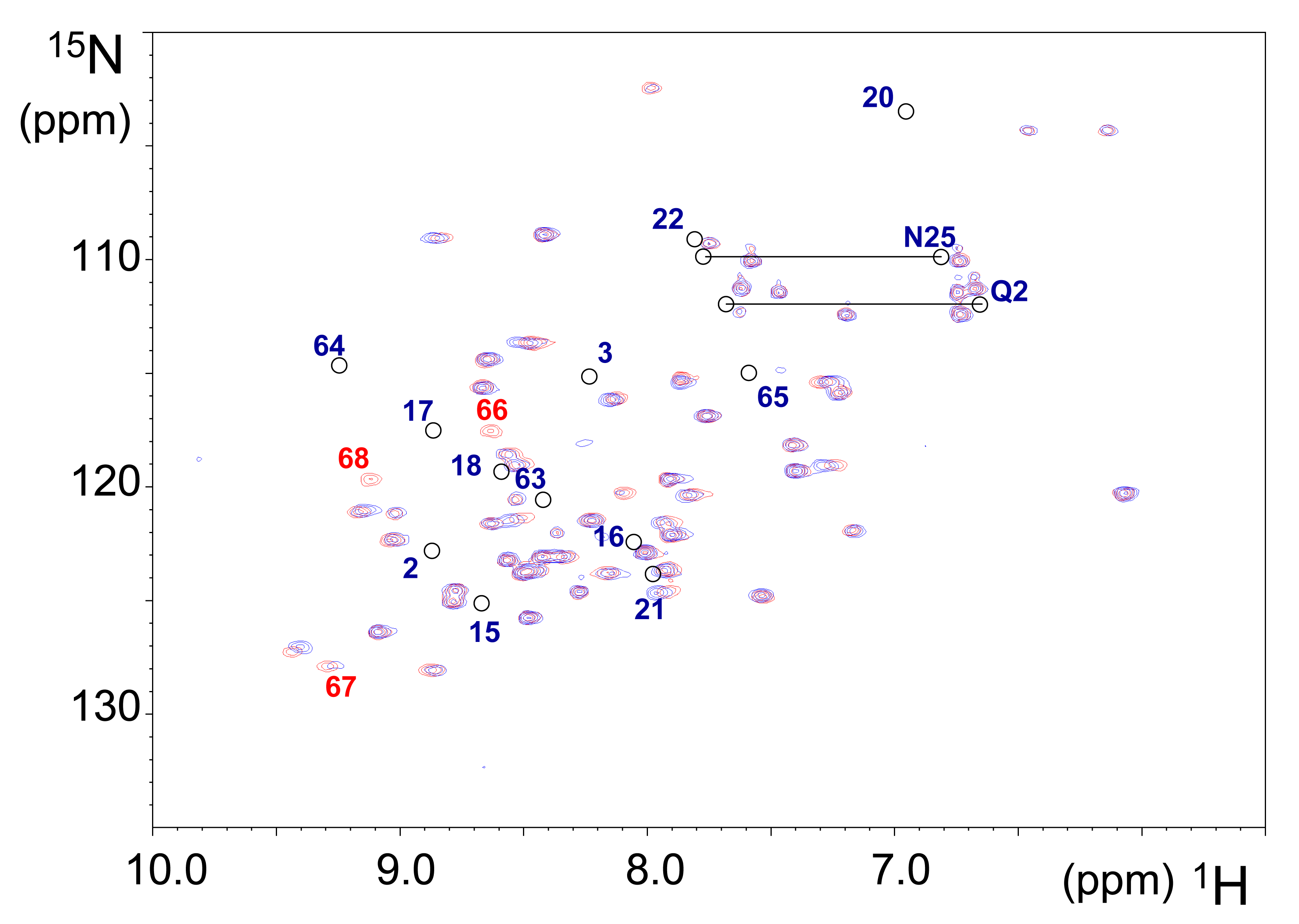

Supplement: Figure S1 — Paramagnetic Cu(II) broadening effects in NMR spectra of Ub. Overlay of 1H,15N HSQC spectra of Ub in 50 mM ammonium acetate buffer at pH 6.5 with one (red contours) and three (blue contours) mol equiv of Cu(II). Backbone amide cross-peaks and side-chains of Gln/Asn residues that disappear upon addition of one equiv of Cu(II) are indicated with circles and labeled in blue; cross-peaks that disappear after addition of three equiv of Cu(II) are labeled in red. (0.29 MB TIF) [file pone.0007052.s001.tif]

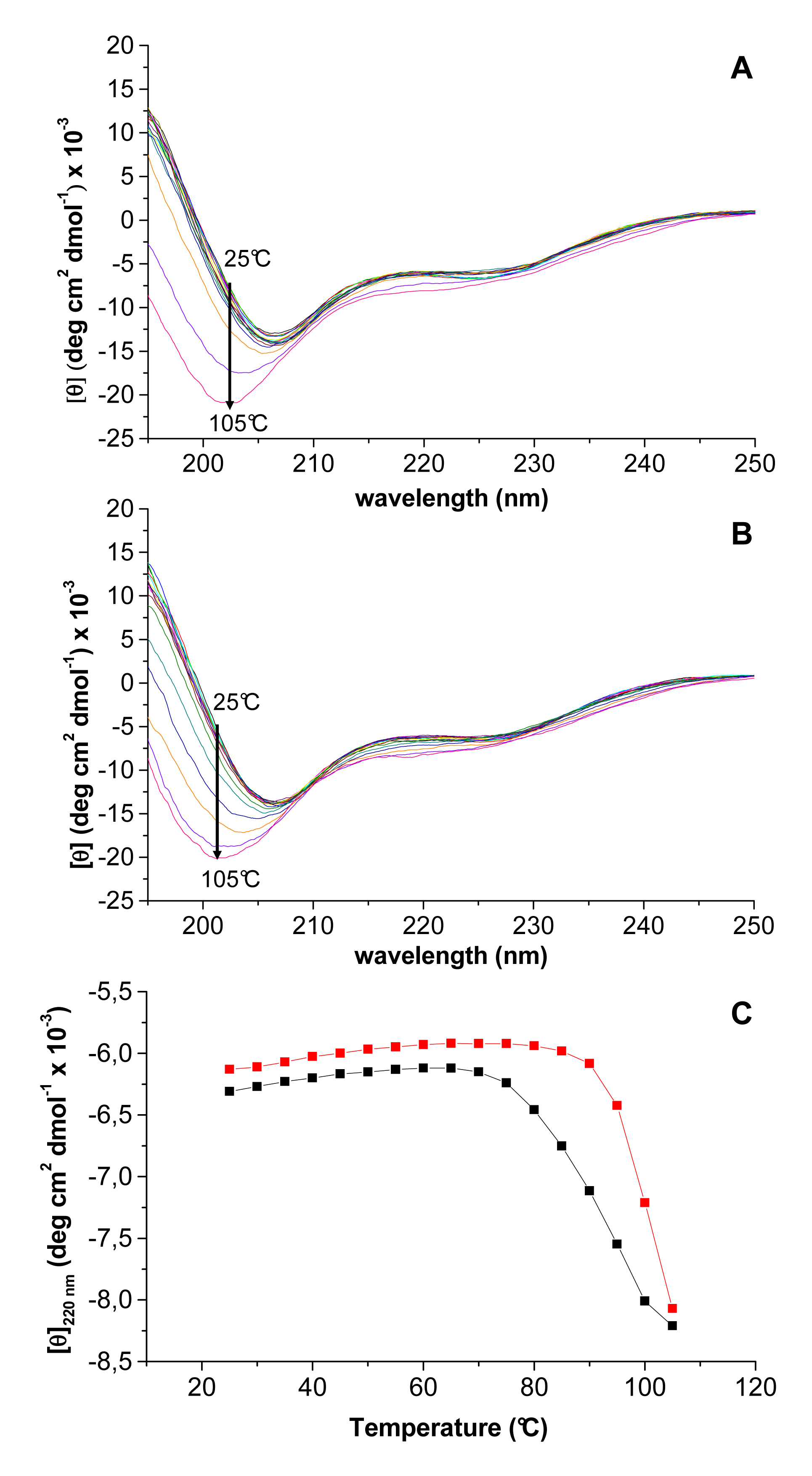

Supplement: Figure S2 — Effect of Cu(II) on thermal denaturation of Ub. Far-UV CD spectra of Ub in the absence (A) and in the presence (B) of one mol equiv of Cu(II) recorded at increasing temperatures from 25 to 105°C in steps of 5°C. The plot of molar ellipticity at 220 nm vs. temperature is shown in (C) for Ub in the absence (red curve) and in the presence of Cu(II) (black curve). (0.43 MB TIF) [file pone.0007052.s002.tif]

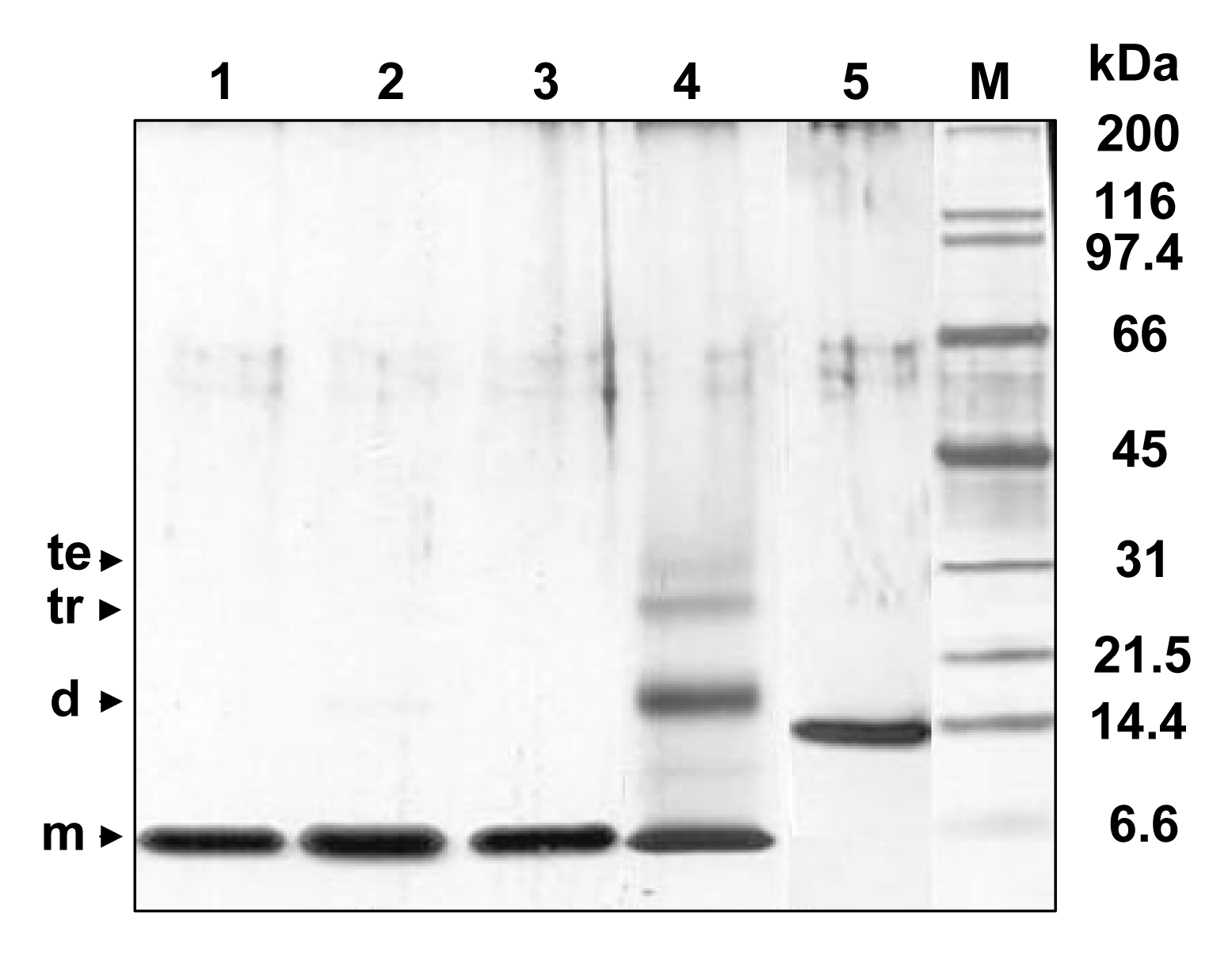

Supplement: Figure S3 — Comparing the effect of Fe(II), Ca(II), and Cu(II) on Ub oligomerization. SDS-PAGE of Ub incubated for 2 weeks at 37°C with 3 mol equiv of Fe(II) (lane 2), Ca(II) (lane 3), and Cu(II) (lane 4). Control experiments were performed on Ub incubated for 2 weeks at 37°C in water in the absence of metal ions (lane 1) and on hen egg white lysozyme incubated for 2 weeks at 37°C with 3 mol equiv of Cu(II) (lane 5). (0.43 MB TIF) [file pone.0007052.s003.tif]

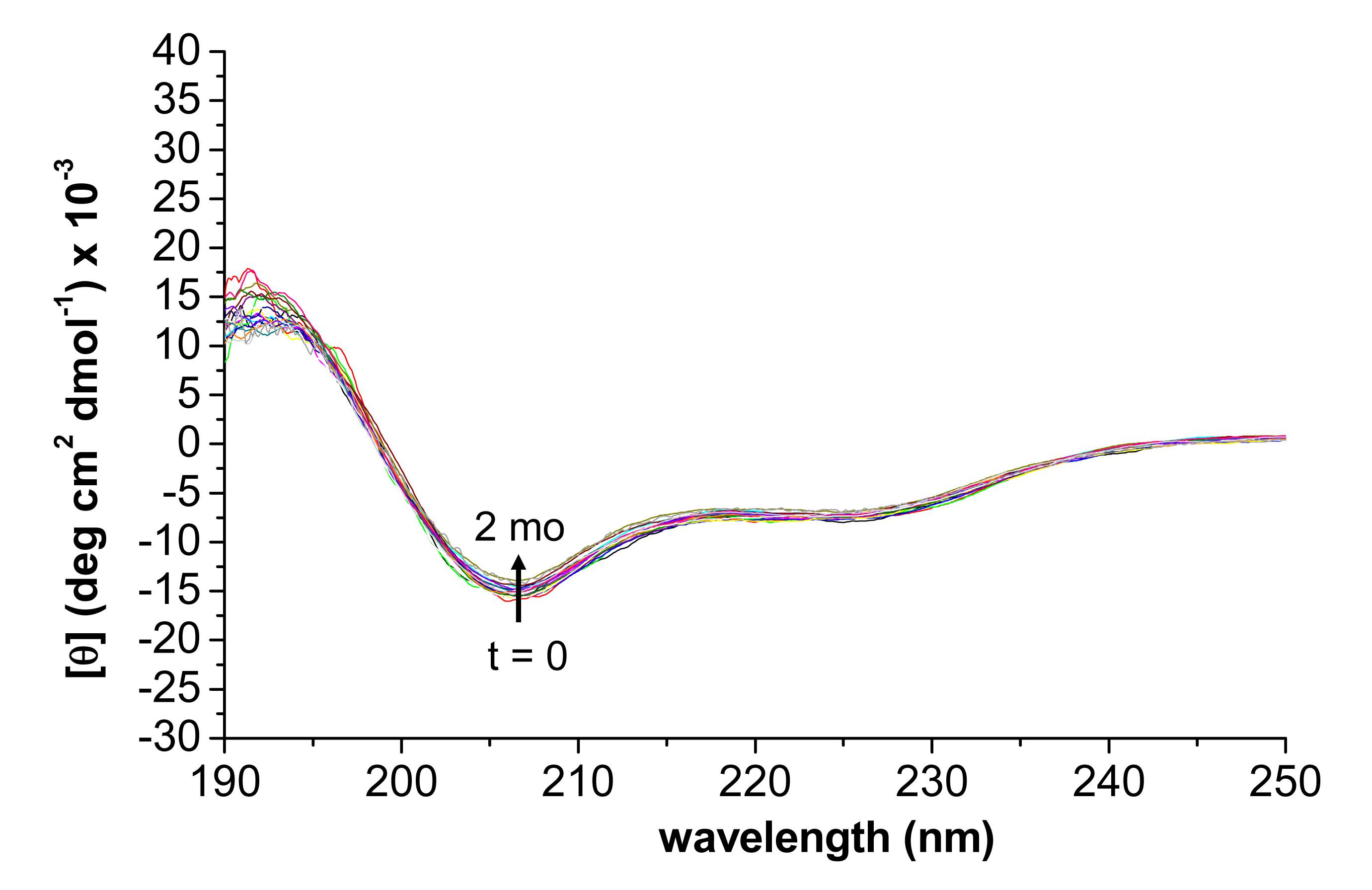

Supplement: Figure S4 — Effect of Cu(II) on secondary structure of Ub. Far-UV CD spectra of Ub incubated at 37°C with 3 mol equiv of Cu(II). Spectra were recorded at different incubation times over a period of two months. (0.28 MB TIF) [file pone.0007052.s004.tif]

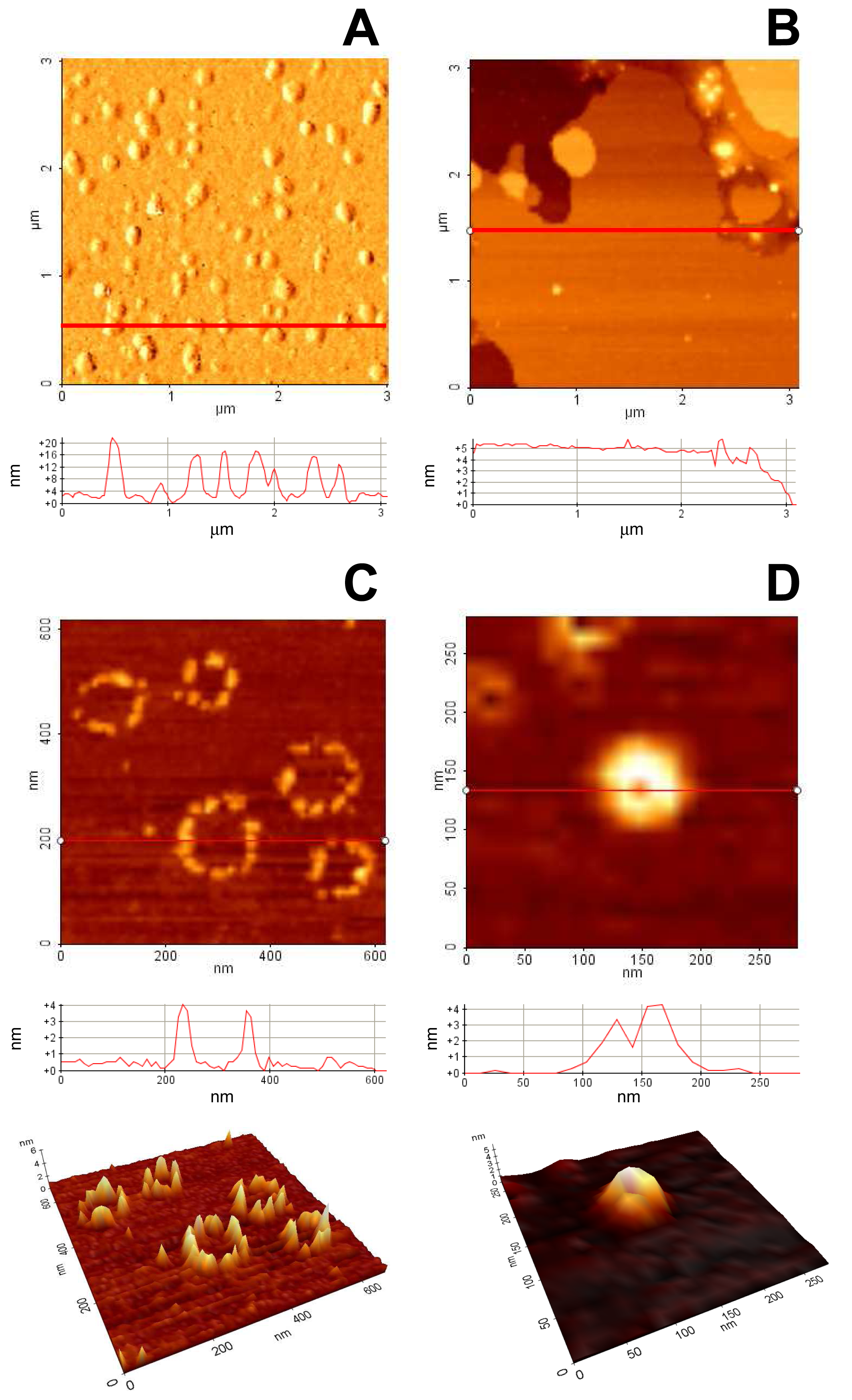

Supplement: Figure S5 — Reconstitution of Cu(II)-stabilized Ub oligomers in phospholipid bilayers. Phase-mode AFM images and cross-sectional profile, taken along the red line, of POPC liposomes (A) and POPC planar bilayers (B) in the absence of protein; topographic AFM images of annular (C) and pore-like structures (D) formed by Ub preincubated for two weeks at 37°C with 3 mol equiv of Cu(II) in aqueous solution and then dissolved in POPC liposomes and in POPC planar bilayers. The corresponding cross-sectional profiles and 3D views are shown at the bottom. (3.31 MB TIF) [file pone.0007052.s005.tif]
